# Family Care for Children with Disabilities in Czechoslovak Documentaries in the 1960s and the 1970s

**DOI:** 10.1177/16118944241287720

**Published:** 2024-10-07

**Authors:** Victoria Shmidt

**Affiliations:** Centre for History of Science 27267 , Karl-Franzens-University of Graz, Austria

**Keywords:** Deprivation, patriarchy, intersectionality of race and disability, socialist Czechoslovakia, public campaigns for family placement

## Abstract

This article discusses the approaches to family care for children with disabilities depicted in documentaries produced by prominent film directors in the 1960s and 1970s, the period explored as re-establishing patriarchal order in socialist Czechoslovakia. Interpreting the documentaries in the context of public campaigns on child welfare reveals mental deprivation as a central concept that brought together the biopower of institutions and patriarchal values. The success and dissemination of Deprivation Theory is shown to have resulted in multiple internal contradictions in the medicalization of public care for children that was initiated in the late 1940s. The article traces the grounding of the dichotomy of public care versus family care by re-constructing the social ideals around child development and family care. These ideals remain one of the obstacles to the deinstitutionalization of care for children with disabilities to this day.

In 2010, the Czech Ombudsman of Human Rights, Otakar Motejl, attacked the deeply rooted practice of restricting substitute family care^1^ for children with disabilities and children of Romani origin^2^:

The question has arisen as to whether, when including children in the Register of Children Suitable for Foster Care and Adoption, there is no distinction between children mainly based on ethnic origin and disability. The suspicion that such treatment is taking place is mainly justified by the Ombudsman's findings from systematic visits to institutions for the provision of public care.^3^

To attack this practice, Motejl repudiated the multiple and overlapping concept of the ‘suitability’ of the child (*vhodnost ditěte*), applied in the regulations on family placement since the 1970s. In the Ombudsman's Statement, the children's health, according to the Law on the Family (2009), should not represent ‘an insurmountable obstacle to placing the child with a family’.^4^ In accordance with the purpose of best care, the ‘suitability of children’ should be consistently rejected as a criterion of placement and replaced by the ‘suitability’ of the substitute family, able to take care of these children.^5^ The blatant insufficiency of this campaign and other current efforts to prevent domestic and institutional violence against ‘other’ children^6^ calls for a historically informed understanding of the obstacles to implementing the right to family life for children with disabilities.

The taboo of placing disabled children in families is a substantial problem in many countries.^7^ I examine the Czechoslovak case through the lens of the transnational historicization of Attachment Theory. This theory places the parent–child dyad at the centre of the exploration of norms and pathology as an essential driving force in reproducing patriarchy since the 1950s—by re-establishing surveillance over women, urging them to prioritize the role of mother and a particular way of enacting it in favour of the proper development of children.^8^ Understanding patriarchal power as ‘the gendered parasitical appropriation of the bodies of others’,^9^ I explore maternal deprivation, namely, the multiple shortcomings in child development due to the absence of a mother's care, one of the main implications of Attachment Theory, as an extreme form of gender essentialism that blocks civic participation in order to overcome utilitarian views on childhood and parenthood.^10^ The attribution of a priority role to maternal care significantly constrained the formation of biosolidarity, the process through which biosocial actors, including parents of children with disabilities, both substitute and biological, perform acts of advocacy on behalf of their biosocial community.^11^ Presenting maternal deprivation as a universal explanatory argument for surveillance over parents (especially mothers) reinforced multiple hierarchies of children and families as more and less suitable for the mission of family care across the globe.^12^ Following Hunnicutt's approach to the historicization of patriarchy as a multi-layered phenomenon,^13^ I examine how suitability for family care became enmeshed with ableism and established the terrain of expert power that placed children with disabilities on the lowest rung of these hierarchies in socialist Czechoslovakia.

Embedding Attachment Theory in the longue-durée of the utilization of motherhood by different political forces contributes to the historicization of patriarchy by reflecting ‘how time and place both contextualize and problematize our thinking about women’.^14^ The role of Attachment Theory in restoring the patriarchal order and aggravating institutional violence against children since the 1960s, including those with disabilities, has not yet received critical consideration. The acceptance of Attachment Theory by the Czechoslovak public stems from notions of preserving the family as a bulwark of freedom against the authoritarian regime and rejecting the historicization of population politics in terms of patriarchy as irrelevant to the socialist period.^15^ Important recent insights, aimed at critically revising the population policy of socialist Czechoslovakia, have problematized the role of experts in the conservative backlash of family policy in the 1960s and its contemporary echo.^16^

The consistent historicization of patriarchal practices calls not only for a deconstruction of their agents but also for an exploration of the trajectories through which alternative discursive practices have been developed. This task is essential for the historicization of disability. Zombie-socialism, ‘a hybridization of ritualistic anti-communist incantations and a neoliberal doxa’,^17^ which attributes the origin of all the contemporary challenges of social welfare to the communist past, still resonates with the multiple shortcomings in understanding the specifics of social policy, including measures targeted at people with disabilities.^18^ Multiple strategies of historicization that aim to deconstruct a homogeneous vision of the socialist past, in favour of a more historically accurate understanding, nuance the impact of Soviet politics on the politics of disability in different Central and Eastern European countries by: (1) unpacking the notions of socialism, communism, and totalitarianism^19^; (2) revising the changes and continuities in socialist social politics regarding previous periods^20^ and (3) extending the geopolitical notion of socialism and connecting socialist Europe with socialism in Africa, Latin America and Asia.^21^ These types of narratives keep the focus on the policies of states and global stakeholders, while redressing past injustices and conquering prejudices against people with disabilities. They call for recognizing the history of civil participation, including its contention with state politics at different levels.^22^

In the first part of the article, I contribute to this process by nuancing the attempts to present family care for children with disabilities between the 1960s and 1970s as determined by two interrelated trends: the medicalization of public care and consistent familialization, which experts legitimized through the concept of deprivation. The reports prepared within the Ministry of Health regarding the reform of public care for young children collected in the National Archive (NA)^23^ shed light on the role of experts and practitioners in conceptualizing issues of childcare in terms of mental deprivation, an umbrella concept introduced by medical experts for explaining various issues of mental development, especially the placement of children in an environment that lacks proper stimulation for such development, such as residential care institutions. The internal reports developed by the analytics of Radio Free Europe and collected at the Vera and Donald Blinken Open Society Archives (OSA) offer additional evidence of anti-communist propaganda as a driving force behind the public discourses regarding family care in socialist Czechoslovakia. By exploring publications in *Vlasta*, the official periodical of the Czechoslovak Women's Union (Československý svaz žen*)*, often seen as an agent and a structure for re-establishing patriarchal order^24^ and ableism,^25^ I contextualize public campaigns that conveyed messages regarding family care for children with disabilities.

In the second part of my article, I interpret the theory of deprivation as one of the most extreme manifestations of child fundamentalism, that is, formal and informal discourses that mobilize the figure of ‘the child’ as an absolute discursive category ‘with which one cannot disagree’.^26^ I follow the options for, and limits to, challenging ‘child fundamentalism’ by rejecting the idea of ‘the child as a nostalgic object from the perfect past and as a symbol of a utopian future’^27^ in films aimed at involving people in the ‘proper’ practices of bringing up young children. The main sources informing this analysis include four documentaries produced in the genre of the film-questionnaire or *film-anketa*,^28^ in which the audience gains access to the multiple opinions and experiences of families motivated to help to children, including those with disabilities. In line with Fredric Jameson's stance on the priority of interpreting film as an agent and a structure of politics,^29^ I explore these documentaries as cultural texts that consistently shaped social ideals of family care by practising a particular cultural politics of affect^30^ around children, including those with disabilities. Two films by Kurt Goldberger, *Děti bez lásky* (*Children without love*, 1963) and *Lidé* (*People*, 1964) represent the central media in the campaigns targeted at recruiting substitute families. I consider these films to be some of the most consistent manifestations of renewed patriarchy. Furthermore, I explore their unprecedented success from the point of view of cruel optimism, one of the strategies of pop culture aimed at disciplining the imaginary about what a ‘good life’ is and how proper people act to achieve it.^31^ Lauren Berlant, the author of the concept, defines affective structure of optimism as internally conflicted. On the one side, it ‘moves you out of yourself and into the world in order to bring closer the satisfying *something*’.^32^ On the other hand, being ambitious, optimism ‘might feel like anything, including nothing: dread, anxiety, hunger’.^33^ This contradiction inclines to return to the phantasies and to ignore rational calculation. The contradiction transforms in cruel optimism when an object of desire that evokes a sense of possibility actually makes it impossible to achieve.

*Čekají každou neděli* (*Wait every Sunday*, by Václav Táborský, 1962), and *Dobrý člověk ještě žije* (*The kind man still lives*, by Otta Bednářová and Marie Šolleová, 1970) utilize the liberal imagination to reshape attitudes towards children and their needs by moving beyond normative feelings and revising the idea of affection.

## 1. The interplay of patriarchy and ableism

### Controversies in medicalizing public care for young children in the 1950s

In the early 1950s, residential care for young children, which had been under the purview of the Ministry of Social Affairs, relocated to the Ministry of Health.^34^ Infant homes and children's homes for children under 3 started to operate as public health institutions^35^: instead of social welfare officers, chief paediatricians began leading these institutions, and instead of educators (*pěstounka*^36^), daily care for children was provided by nurses. Those educators who wished to continue their employment received medical retraining,^37^ maintaining the interdisciplinary, medico-pedagogical profile of their activities.

Institutionalizing the priority of medical care for young children reinforced the long-term operational differentiation of people with disabilities according to their ability to be remedied, trained, and socialized.^38^ Early childhood began to be seen as a unique period in which to ensure an optimal development trajectory by uniting educational and medical aspirations. Since the late interwar period, prioritizing medical expertise in the organization of care for children triggered multiple, bottom-up initiatives, including long-term observation, focused on the development of children in different circumstances, including residential care. After 1945, large-scale initiatives targeted at supporting the mental development of young children in institutions shaped the longue-durée of medical experts. This priority—associated with the growing influence of social paediatrics—was a response to the call for a combination of nature and nurture, which was essential in legitimizing socialist public health as authentic and sensitive to people's needs.

In 1954, the chief regional paediatricians issued their recommendations for further improvements to support for children's mental development (*neuropsychický vývoj*). The recommendations reflected very early attempts to prevent hospitalization by making contact between the child and the caregiver more sustainable: ‘one nurse should be responsible for the one group of children made up of no more than 7 to 10 children and make records about their mental development’.^39^ Options were developed for mothers to stay with children who needed to be placed in institutions because of medical issues.

At the beginning of the 1950s, public care, along with intensive institutionalization, began to be politicized, with both communist and anti-communist propaganda actively using conflicting ideals about raising children to their advantage. The medicalization of childcare was part of the prioritization of prevention within welfare politics, which began in the late 1940s with the nationalization of public health. This was aligned with the Soviet model developed by Nikolai Semashko, which aimed to provide universal access to healthcare^40^ and was legally implemented through the Unified Preventive and Curative Care Act (*Zákon č. 103 /1951 o jednotné preventivní a léčební péčí*). The Sovietization of public health was attacked through anti-communist propaganda and criticized from the position of patriarchal values. Anti-communist propaganda emphasized the coercive methods used in the raising of children by the state.^41^ This point resonated with the no less coercive methods that involved ‘their mothers working 13 hours daily’ and upon ‘whom the positive image of public care was imposed, while workers could not avoid placing their child in children's homes [and] while they were not happy about entrusting their offspring to foreign people, [they] had to do it’.^42^ The inability to ensure standards for children's well-being in the system, due to the many shortages of material and human resources, was often mentioned with regard to the spread of infectious diseases and the poor state of children's health.^43^

The entirely patriarchal motive of alienating women from their ‘natural’ desire to provide care for their children was accompanied by the discursive practice of blaming irresponsible mothers. Since the 1960s, blaming mothers became one of the pillars for prioritizing substitute family care over the reunification of biological families. Unsurprisingly, in the reports prepared by Radio Free Europe,^44^ the image of the woman who preferred to shed the burden of caring for a child was connected with the ‘wives of Communist Party bosses who do not need to work but place their children in order to slack off on caring for them’.^45^ The view on the placement of children in institutions as sacrificing them to ‘improper’ social ideals or selfish interests resonated with presenting the despair of working mothers unable to help their offspring. An emotional statement by a mother, relayed to Radio Free Europe by a runaway neighbour, asserting that ‘it would be better if the children had not been born at all, than having to start their lives by being placed in an state childcare’,^46^ resonated with multiple unflattering comparisons between Czechoslovak family policy and Chinese family policy,^47^ which, in anti-communist propaganda, reflected a structural violence against family life.^48^ The clash between communist and anti-communist propaganda inclined socialist ideologues to adopt the patriarchal pathos of anti-communist propaganda in the next decade, when the political climate was liberalized and the prerequisites for further consensus between the communist authorities and the public began to be established.^49^

However, the rhetoric of rescuing children from institutions had no resonance when it came to children with disabilities. The institutionalization of care for them did not become the subject of criticism—on the contrary. The obvious lack of residential care institutions for children with disabilities was seen by practitioners and acknowledged by propagandists on both sides as one of the main challenges for the welfare system in post-war Czechoslovakia. Along with strengthening assessment and intervention, physicians stressed the necessity of eliminating the joint education of healthy children and ‘retarded and abnormal children’, and eliminating the reasons for placing ‘normal’ children in children's homes, which meant more consistent cooperation with families.^50^ Later, these recommendations were implemented at the national level.^51^ As a result, public care started to become a customary option for these children. In the first decade after the Second World War, there was a proliferation in many types of institution for children with disabilities: the number of schools for ‘handicapped’ children increased more than fivefold, from 130 in 1945 to 651 in 1959, and the number of children placed in these schools increased from 8,011 to 39,764. Most of these institutions were special boarding schools with reduced curricula,^52^ and the grounds for a hierarchy of children more and less suitable for family care were established. Alongside the massive dissemination of the idea of maternal deprivation, this hierarchy offered new options for development within the improving economic conditions and the possibility of providing for the basic needs of children.

### Maternal deprivation and re-establishing patriarchy

The Czechoslovak pioneers in promoting family care as a form of prevention of mental deprivation (*deprivační syndrom*) were paediatricians. They were often the heads of infant homes, so they were very familiar with all the formalities of placing young children in institutions. Marie Damborská,^53^ Antonín Mores^54^ and Ladislav Zeman^55^ were the most prominent representatives of this cohort, which introduced methodical and organizational innovations that mimicked the conditions of family care in the second half of the 1950s.^56^ They also developed research projects aimed at confirming the destructive impact of placement in institutions early on in childhood development.

Positing public care as merely supplementary to family care became a mainstream view among the practitioners:

Children's homes should only serve as temporary placements, in no case permanent ones. Will family conditions improve to such an extent that the child can be returned to the care of the parents after a certain period? Or will they not improve? It is up to the managers of the children's homes to take care of the child's adoption.^57^

Since the early 1960s, family placements for children in institutions, especially those for children younger than 6 years old, became the main criterion for the efficient operation of residential care centres. Short reports by local authorities about the success of family placements in popular periodicals were accompanied by a comparative analysis of the ways in which institutions had implemented these. Miroslav Trubač, a member of the regional Board for Youth Education in Moravia, stressed the role of external networking around children as a factor:

Five-year-old Vojta from one [children's] home, on his way to his new parents, qualified all the vehicles we encountered as an ambulance with a doctor—because he didn't know anyone else, no one else went to their home. On the other hand, three-year-old Mirek, from another home, had already identified a bus, a loading truck, a bicycle, and a motorbike on his way to his new parents. Both boys are *perfectly healthy* [my italics], only each from a different home.^58^

The moral and social obligation to adjust the life and the environment of residential care institutions so that they resembled a well-balanced family as much as possible^59^ was challenged by sorting children into those more or less ‘suitable’ for family placement because of their former experiences, including residential care. ‘Every child who is not loved is abused’—this imperative to love, attributed to Jíři Dunovský,^60^ “becomes an imperative to extend the ‘ideal’^61^ sought by medical experts to parents, especially mothers, who ‘can’ return the ideal to children:

The family is the only space where a child can fully satisfy their intense emotional needs, receive recognition of their uniqueness and irreplaceability, and establish deep and lasting affective relationships and acquire a sense of deep security.^62^

This is classic Attachment Theory, as conceived by John Bowlby, which is focused on different types of motherhood as factors in the development of constructive or destructive attachment. Czechoslovak psychologists Zdeněk Matějček^63^ and Josef Langmeier^64^ also highlighted the child's experience of neglect and abuse as central in explaining stubbornly regressive behaviour and development.

Consistent comparisons of the child with a plant and the educator with a gardener, well-known since the Enlightenment, inclined the enthusiasts of Deprivation Theory to contrast the vital environment of the family with the artificial ‘greenhouse’ environment of residential care institutions. The idea of balance became the key to a strategy of preventing deprivation. Matějček and Langmeier described the ideal role of the family: parents were to develop a ‘dynamic but unified’ environment throughout childhood that would provide a feeling of ‘home’. The very possible lack of such dynamics and stability was defined as a ‘misdeed against a child’, and the idea of psychological surveillance aimed at recognizing children at risk of deprivation remained central in the majority of the texts written by these authors: ‘[T]he current purely medical dispensary will also be accompanied by a psychological one; it will be possible to catch children who are potentially ‘at risk’ and then give them special preventive attention’.^65^

The call for steady control—the flip side of idealizing the family—translated into selection procedures for those children considered too ‘other’ and too much of a social risk for family placement (*společenská únosnost rizika umístění dítěte*). Children of ‘other races such as Gypsies or mulattos’, as well as children with disabilities, were socially defined as being not only visually different but *zvláštní nehezké* (especially unpleasant). Because they did not represent the desired cultural reflection of society, their *nápadnost zjevu* (conspicuousness) came to be seen as a source of societal volatility. This, in turn, became the main obstacle to their family placement, and especially to adoption.^66^ Hereditarily conditioned deviations were seen as another obstacle, often in combination with ethnic ‘otherness’. By the early 1960s, placing children with disabilities or children of Romani origin—who, it was believed, ‘would be difficult to understand and who do not respond to adult expectations’^67^—into institutions from the very beginning of their lives was common practice. As a result, these children were subject to double labelling: as the ‘other’, but also as unfit for family placement because a long-term stay in a public care institution was considered a risk factor for a successful family placement. At the same time, the predominance of family care began to contest such a univocally negative view on family placement for these children: ‘We believe that the opinion that only the best children can be adopted remains prevalent: i.e., the smartest, healthiest, and unfortunately also the most beautiful. And that is a huge mistake!’^68^

The choice of family placement had to be based on evaluating the child's ‘fitness’ score. The suitability of the child was assessed by three interrelated indicators of deviations from the norm: heredity, early development and social conditions (features of the biological family).^69^ Accordingly, only children who had suffered less from deprivation in the biological family and institutions, that is, those up to 3 years old, continued to be considered suitable for a family placement.

The ‘otherness’ of children not suitable for family placement resonated with the ‘otherness’ of their mothers. Dunovský introduced a straightforward division of these ‘other’ parents into those who could not provide care, those who did not want to provide care and those who had not been taught to be good parents.^70^ Within the public campaigns aimed at promoting proper mothering, which were on the verge of a moral panic, this division had a patriarchal quality to it. Presenting the experience of being placed in an institution as playing an essential role in deprivation produced various forms of interrelated objectification of children and women. The introductory passage to an article aimed at persuading readers of the women's magazine *Vlasta* of the ‘hereditary vicious circle’ of maternal deprivation read as follows:

Heda can't say anything nice about her childhood. Her parents hated each other, and a constant black cloud of quarrels hung over the family. Her father neglected Heda, her mother sometimes took out her resentment towards the father on her daughter, and at other times, she treated her with excessive condescension, and this was even more unpleasant for Heda.^71^

To make Deprivation Theory more accessible, the author introduces introduced three exemplary stories of young women whose immoral and irresponsible behaviour has determined the deprivation of their offspring, with their own childhood deprived of parental love. The first story, *Oklamaná* (Deceived), was about a girl from a ‘socially vulnerable’ family who indifferently had an affair with an African student and who received neither family support nor community care for the child. The second story, entitled *Nerozvážná* (Ill-advised), told tells the story of an ambitious young woman from a working family who lives in a *svobodárna* (dormitory for working women). She gave birth to a child by her married lover, who was also her boss. Her subsequent marriage to a man whose mental qualities, which fell far short of those of a noble-minded man, prevented him becoming a good father to the adopted daughter. And the last story, *Nezodpovědná* (Irresponsible), told the story of a ‘hereditary’ prostitute who, with her falsely innocent appearance, not only deceived the expectations of the social services that tried to help her, but also reproduced a vicious circle of debauchery. Each of the stories, narrated in overtly misogynistic language, was accompanied by expert commentary on how such behaviour among mothers had irreversible consequences for the psyche of the child, in fact, results in disability.

Attempts to help biological families keep their children, such as organizing flats for single mothers of special-needs children within infant homes, did not succeed, not least because of such systematically negative and distrustful attitudes towards them.^72^ The belief can be explained by the fact of the disparity between public readiness to accept children and the very fragmented legal regulation of adoption and foster care, which persisted until the early 1970s.^73^ In the eyes of those who took responsibility for children in institutions, the biological parents’ resistance to relinquishing their parental rights and giving the child a chance to be adopted was the main hindrance to timely family placement:

What to say about a mother who refuses to consent to the adoption of her ninth child when the previous eight, both legitimate and illegitimate, are in state care. At the same time, the district national committee informs us that the mother is living a scandalous and disorderly life.^74^

While in the 1950s, the medicalization of care for pregnant women was accompanied by the perpetuation of the discourse around a mother's responsibility for the ‘innate’ health of the child, attributing blame to mothers for the birth of a child with a disability, Deprivation Theory held mothers responsible for ‘acquired’ diseases, often defined as *vývojová poškozenost prostředím* (impaired mental development due to the environment). Judith Bennett has called such continuation the ‘patriarchal equilibrium’, when ‘the force of patriarchy was undermined in one sector but was subtly countered by responses in other sectors’.^75^ The concept of deprivation with its emphasis on the opposition between residential care as ‘unnatural and against biology’ and family care as the natural default state, re-established patriarchal power not only over biological processes but also over the knowledge of these processes and its dissemination.^76^

In the 1960s, documentaries became one of the primary media channels for broadcasting ideas about family, childhood and the politics of care. In addition to portraying ‘proper’ and ‘improper’ parenthood based on popular texts about attachment and deprivation, these documentaries educated the public through the embodied experiences of the films’ main protagonists, namely, children and their parents. I explore how four films aimed at promoting family care for children, including those with disabilities, stirred up the emotions of the public^77^ and either reinforced or problematized the patriarchal order.

## 2. Family placement of disabled children in documentaries

### The cruel optimism of the (im)perfect child in the perfect family

By presenting a completely negative perspective on the inner life of children in institutions, Kurt Goldberger, the director of popular science films, makes ‘a persuasive but also a more appalling argument’^78^ in an attempt to shape the negative image of residential care:

Children brought up exclusively in a collective do not know what is beautiful, how to love, nor do they know how to work […] The stereotypical life, when yesterday is as tomorrow, leads to a deficit in initiatives, nothing look forward to; they remain emotionally superficial.^79^

His documentary *Děti bez lásky* can be seen as an educational film that promotes desirable forms of behaviour (family care) and increased social awareness of the consequences of undesirable patterns (public care). The combined aim of teaching and motivating viewers through a better understanding of children is reflected in the film's structure. The 43-minute film consists of two parts. The 7-minute introduction invites viewers to a mental health counselling centre, where several children of different ages follow the camera with suspicion. A voice from behind the scenes relays the official record of three cases in which different circumstances have resulted in the children being placed in institutions, with their behaviour then becoming problematic in different ways. Each case ends with the same diagnosis: ‘deprivation’. Next, the audience witnesses two interviews, one with a 14-year-old girl and one with a 10-year-old boy. The children's shameless self-reports of their delinquent behaviour (sexual promiscuity and thievery, respectively) resonate with the powerlessness and uncertainty of the caregivers who made the decision to place these children in institutions while they were infants.

Along with the interviews, viewers are presented with drawings produced by these children as part of a psychological assessment. The series of portraits of families inclines the viewers towards a direct analogy between placing children in residential care and placing them in detention. The introduction ends with an explanation provided by Matějček: ‘In short, the deprivation syndrome is a set of shortcomings [and] abnormality in the child's behaviour which arise from emotional suffering in the first years of life’.

**Figure 1.**
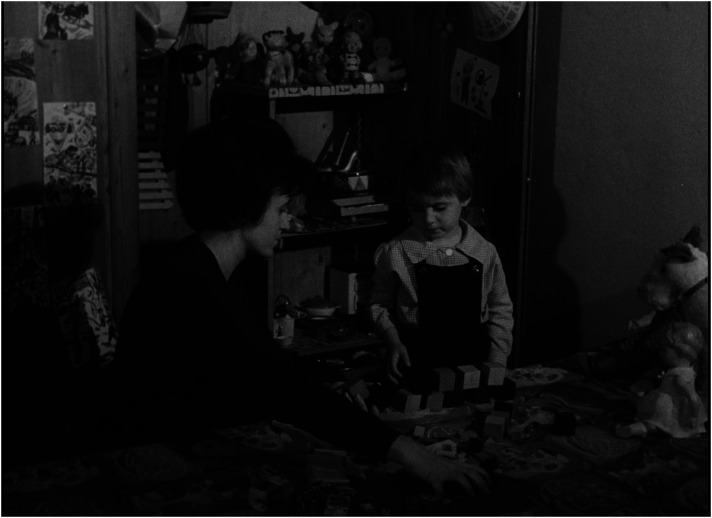
Family child versus collective child (*Děti bez lásky*, 1963). *Source*: NFA.

**Figure 2.**
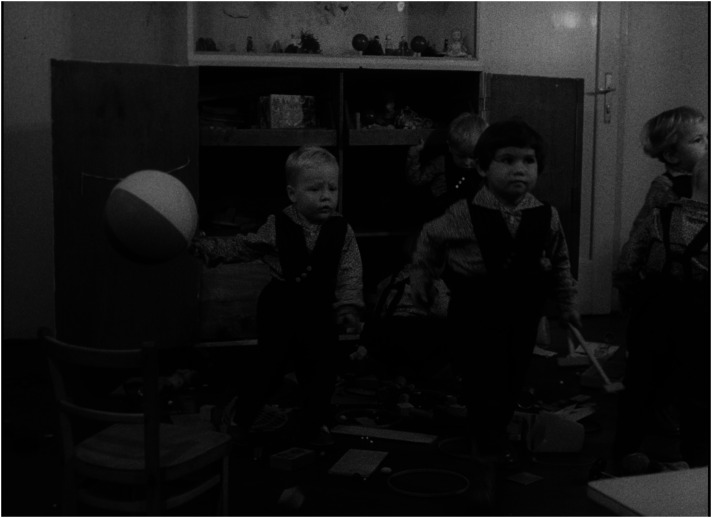
Family child versus collective child (*Děti bez lásky*, 1963). *Source*: NFA.

The main part of the film compares the early development of children in families and institutions at different stages from birth to 5 years. To achieve a sharp contrast between family care and public care, an individual portrait of a child in a family is compared with a group portrait of children in an institution. The tactics chosen to convince the audience of the destructive effect of bringing a child up in an institution are based on the opposition between child and adult that permeated the patriarchal approach to constructing the image of the child in mass culture: ‘Their [children's] expressiveness invites a reciprocal expressiveness from adults who look at them. But together with ecstatic fulfilment comes the threat of total disruption. Childhood poses a challenge to the hard-won stability of adulthood’.^80^ Presenting children in institutions as a group of poorly managed creatures who, in contrast to children in families, do not invent, create, or build but remain passive, reproducing existing patterns and destroying their surroundings, easily inclines the viewer to see them as a ‘savage’ tribe on the verge of delinquency, a threat to civilized society (see Figures 1 and 2). ‘They lack an attitude to value things—not feeling it at five or ten or fifteen’, says the voiceover, a dispassionate commentary that accompanies the play of 3-year-old children who scatter their toys everywhere. If the viewer feels pity for the babies in the orphanage who either cry or suck their thumbs, one can only feel fear and disgust for the children who behave like ‘barbarians’.

The unprecedented enthusiasm with which *Děti bez lásky* was received by the Czechoslovak public was reflected in numerous laudatory reviews:

No article in the most popular magazines, no programme on the radio or on television can surpass Goldberger's new film in eloquence and persuasiveness. Goldberger, with his excursions into the essence of the problem of emotional deprivation among children, following them with his camera both in their family homes or in the dining room of a children's home, is, as an appeal to all people—and decisive social organizations—more important than the most shocking statistics of a psychologist.^81^

The international success of *Děti bez lásky* was undoubtedly one of the driving forces that led to an acceptance and dissemination of socialist theories of deprivation as a strong argument against public care. The film participated in the 25th Venice Film Festival and won the *Targa Leone di San Marco per il miglior film di vita contemporanea e di documentazione sociale* (Lion of San Marco for the best film of contemporary life and social documentation). This win only reinforced the international acceptance of the Czechoslovak approach to promoting family care on both sides of the world divided by the Cold War. In the West, because of the close connection with Bowlby's theory, the Czechoslovak version of maternal deprivation was seen as closely linked with negating Soviet pressure. Peter Frank^82^ presented the efforts of Czechoslovak experts to improve substitute family care as in direct opposition to Soviet collective institutional care. According to Frank, while it was difficult to distinguish between social policies in the Soviet Union, Bulgaria, and Poland, in the treatment of the deprived child, Czechoslovakia was challenging communist assumptions:

Instead of collective institutions—the nursery, the kindergarten or the children's home—being regarded as a panacea for the problem, these are now recognized as having relatively little value in caring for the child who is permanently removed from a stable family environment.^83^

This myth about the anti-totalitarian pathos of Deprivation Theory continues to feed into its uncritical acceptance, making it one of the most powerful legacies of the patriarchal order that calls for consistent deconstruction. One of the most recent examples includes some scenes from *Dětí bez lásky* used in the documentary *Syn nepřítele státu* (*Son of an Enemy of the People*, 2022, by Eva Tomanová) about the fate of Karl, the son of Otto Šling, one of the victims of the Stalinist purges in Czechoslovakia in 1952, whose children were placed in a boarding school after the arrest and execution of their father. This recent documentary follows the dramatic twists and turns in Karl's life, including his divorce in 1968, his escape to the West, and his recent efforts to reclaim from the public archives the letters his parents sent to their children from prison. The film's creators not only put forward Deprivation Theory as a way of interpreting Karl's life as problematic but also connect this trauma with speculation on its transfer to future generations. However, the attempt to apply Deprivation Theory to the task of family placement for children with disabilities questions the utility of such a theory due to its patriarchal view of disability as undesired ‘otherness’.

*Lidé* was filmed 1 year after *Děti bez lásky*. The idea to make this film stemmed from the unusual but successful campaign aimed at placing three children—one Romani girl ‘without known disabilities’ and two boys with visual impairment—in families. The campaign was initiated by Antonín Mores and *Vlasta*. In early 1963, *Vlasta* published a call for adoptive parents for the three children from the infant home in Olomouc. It included their photographs and short descriptions of the children. While *Vlasta* had already published such campaigns for ‘healthy’ children, it was the first time that children with special needs were ‘offered’ for adoption.^84^ Public take-up of the call was unbelievable, even for the journalists and for Mores: ‘It was always about children with various disabilities; one of them was even blind. Is the desire for children be so strong that it is not be intimidated by such big obstacles?’^85^ 150 families responded to the call, and each of the three children was placed with a family.

*Lidé* presents the story of some of these families to stress not only the variety of motivations behind people deciding to adopt children ‘condemned for a flaw in beauty’ but also their common belief in family happiness as a pre-requisite for creating attachments to young disabled children as people with a potential for happiness.^86^ With this focus, Goldberger reproduces a primary mechanism for making optimism cruel, limiting the options for emancipation from imposed affects, namely, imposing the inevitable obligation to become happy through loving.^87^ Parents (and viewers) are captivated, on the one hand, by fantasies about happy childhood that block the acceptance of the child's otherness, and on the other hand, by fantasies about good parenting as guided by the mission of happiness (including the functional health of the child). Propagating such a child–parent relationship perpetuates the work of romantic love as the primary means to happiness. This work is deeply rooted in Western mass culture, which cultivates fantasies that limit female agency through heteropatriarchal expectations.^88^

*Lidé* begins with a gallery of children's portraits—close-ups of seven toddlers with unhappy and even tearful faces who are described as sentenced to ‘permanent solitude because of their otherness’. Four of these children are obviously not ‘white’ and disabled; the three ‘white’ children have different disabilities. Whereas *Děti bez lásky* is based on the opposition between family care and public care, the main aim of *Lidé* is the multiple differentiation of children and families in order to answer the question: ‘What is the most suitable adoptive family for a child no one wants?’ Shifting the focus from the suitable child to the suitable family opens the door to profiling desirable and undesirable families for adopting less than ‘perfect’ children. With a focus on the crucial role of the family, the film reflects research, begun in the late 1960s, which traced the social trajectories of people from infant homes who had reached adulthood, including those disabled and of Romani origin.^89^ The film focuses not only on parents but also on the family as ‘a real home, the only place in the world where a wonderful feeling of security reigns’. It furthermore asserts that ‘a man is born wanting nothing more or less than an ordinary life’.

*Lidé* unpacks the universality of family space or ‘a particularly strong sense of family life’ (*zvláštní silný smysl pro rodinný život*) by portraying families in which the mutual proximity of family members is represented through intensive positive communication. While the film stresses different social backgrounds, ideals and motivations, emotional ties among family members remain central to visualizing these families. The film directly connects a well-operating system of interpersonal boundaries with intra-family agreements that help to maintain emotional stability. For instance, in multigenerational families, the decision to adopt a child is discussed between spouses and then with children and grandparents, including the distribution of the tasks necessary to the care of the adopted child.

**Figure 3.**
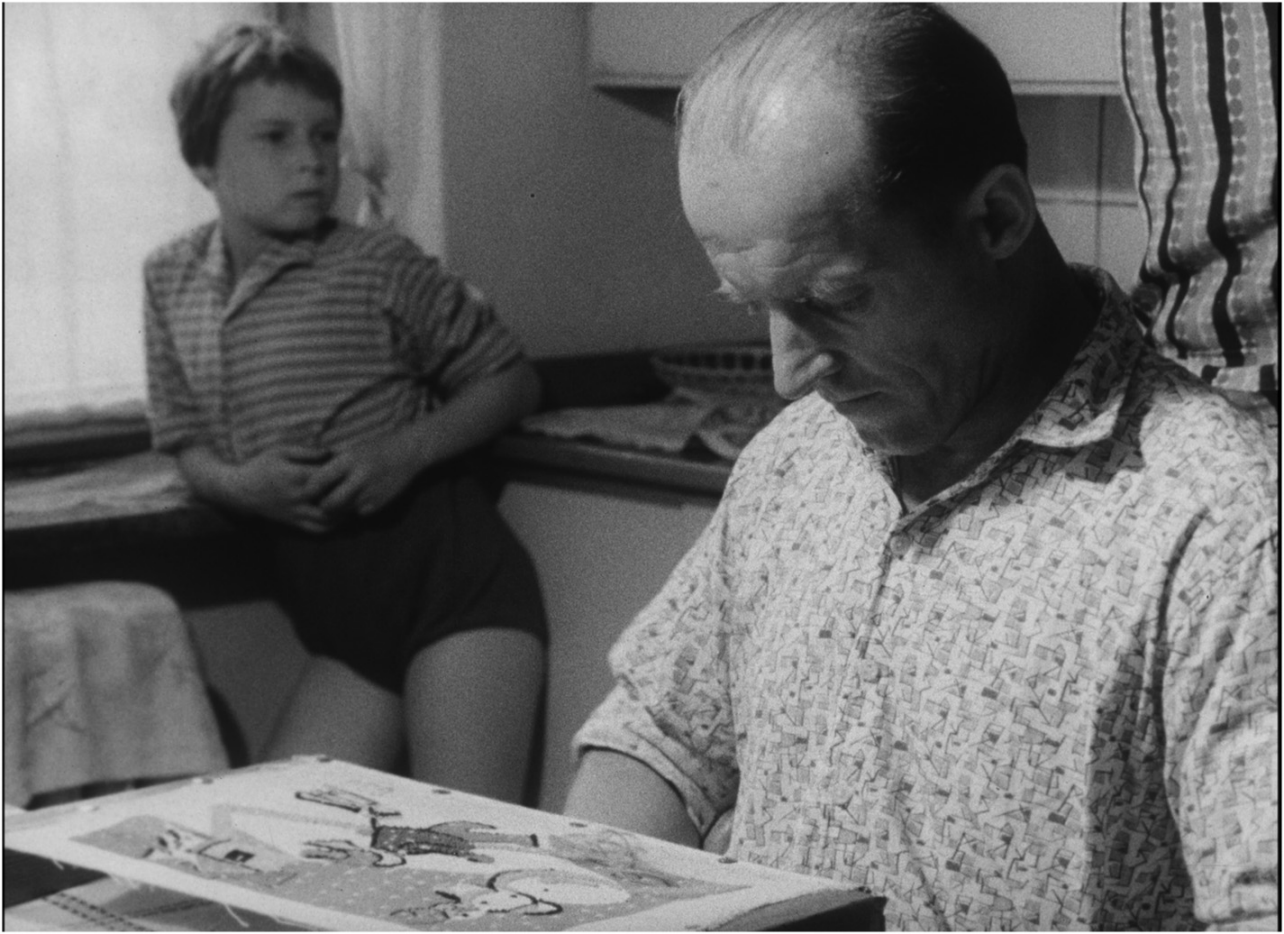
Fathers of perfect families (*Lidé*, 1964). *Source*: NFA.

**Figure 4.**
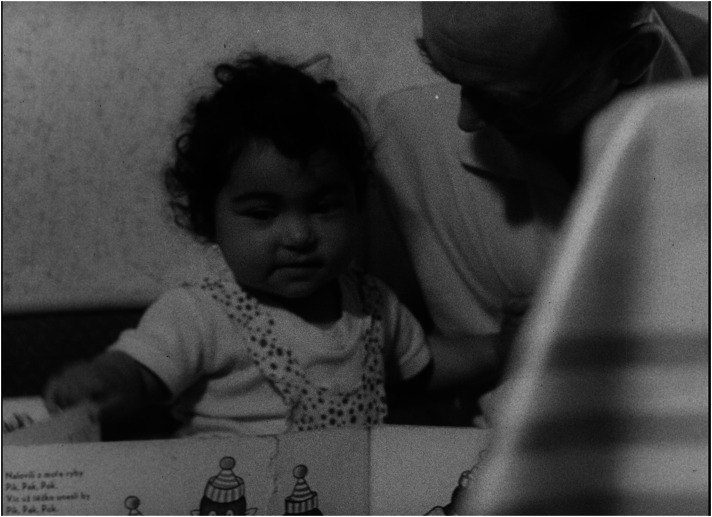
Fathers of perfect families (*Lidé*, 1964). *Source*: NFA.

**Figure 5.**
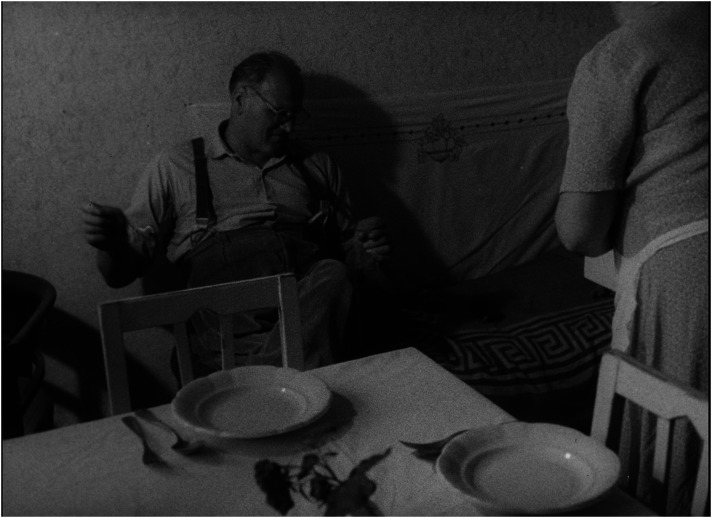
Fathers of perfect families (*Lidé*, 1964). *Source*: NFA.

**Figure 6.**
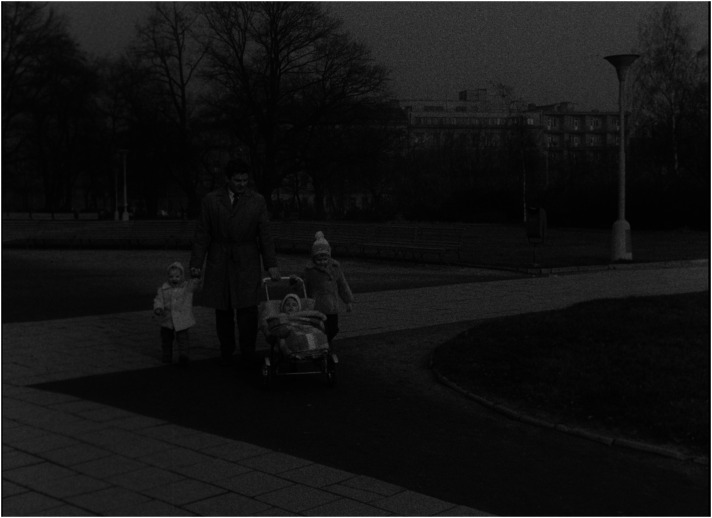
Fathers of perfect families (*Lidé*, 1964). *Source*: NFA.

In a consistent way, *Lidé* advocates a redistribution of responsibilities in favour of the father's involvement in childcare as a main pre-requisite for the ‘healthy’ operation of families. Each of the exemplary families includes a portrait of the father. Foster fathers, darn socks, wash nappies, and then walk with the children, feed them and, instead of going to the pub, even choose embroidery as a hobby in order to spend more time with the family (Figures 3 to 6). Along with stressing the unique role of a mother's love, this pattern aims to legitimize parenting as the ultimate life goal for all.

The film suggests that emotional proximity is a necessary but insufficient condition for the successful placement of children with disabilities in substitute families. Implicitly, *Lidé* questions the utility of the resource of emotional proximity by constructing an ambivalent status for disabled adults who have expressed the desire to adopt a disabled child. Two of the three families who intend to adopt blind Petřík have one parent with disabilities, but this parent has managed to live a full life: becoming a parent, a specialist in childcare, and having a steady income. At the same time, these potential adoptive parents cite their experience of being in an institution and the lack of family care in childhood as challenges that they do not wish on anyone. Notwithstanding, these people do not gain the right to bring up the boy; the family chosen for placement consists of young, healthy and ambitious parents with two healthy children and a grandmother. This choice reiterates the patronizing trope of stressing the multiple vulnerabilities of disabled children and the need for protection from this vulnerability that were typical of media texts at that time.^90^ The grandmother has taken on the main task of educating Petřík—with considerable help from a boarding school for children with visual impairments, where she learned how to support the development of a child. Even she admits that ‘if his mental development does not improve, there is always the option to be placed into an institution’. The story of Petřík's adoption ends with the background comment: ‘They [all three children] will learn to think and to love; the childhood she [the grandmother] is guarding and protecting is a good childhood, in which a child learns to be a human’.

An off-screen commentary at the end of the film directly links the task of adopting children with disabilities with the mission to repair all of society. While Petřiík‘s new family walks slowly down the street, the audience is asked the question: ‘Will we achieve our great goals if we are people who do not learn to stop and understand if someone around us needs help?’ Aligned with cruel optimism, this turn from an ideal of family love to an ideal of community support establishes the ‘affectively stunning double bind’ of family placement for children with disabilities, firstly bound to fantasies about normalizing ‘abnormal’ children that block the allocation of family placement and secondly bound to the optimism promised by the socialist society, which remained a representative fantasy.^91^ Repeated images of adopted children and their parents smiling and laughing reinforce this message. What was the politics of affect in films that deliberately deconstructed the oscillation between the sacralization and demonization of the family in favour of family arrangements for children?

### Family placement as civic emancipation in politically unreliable documentaries

In the early 1960s, driven by personal motives and supported by friends,^92^ Václav Táborský produced three films, no longer than 12 minutes each, designed to shed light on the crisis in family life. *Dva stoly mezi námi* (*Two tables between us*, 1961) presents the internal monologues of two divorcees, using multiple flashbacks to help understand what had caused their marriage to fail. The other two films deal directly with the problems that children face due to family crisis. *Umělci nikoliv nejmenší (Artists not the least*, 1960) depicts the limits and opportunities for gifted children, who only have a chance to develop their artistic talent if they are supported by their family. *Čekají každou neděli* (*They wait every Sunday*, 1962) presents a typical Sunday in an orphanage for children between 3 and 6 years old. It is the day when children are permitted to meet their relatives or potential substitute parents.

This trilogy can be seen as an example of the new documentary movement initiated by the group *Čas*. The creative manifesto of the ‘*časists*’ promised a turn towards truth and authenticity.^93^ In *Čekají každou neděli*, this principle translates into a thoughtful and attentive following of the children's emotions. The subjugation of the whole variety of children's emotions and their manifestations—despair, longing, hope, fear, distrust, feigned indifference—to the expectation of the most important thing for the children, namely, the opportunity to end up with a family, challenges the stereotypical view of residential care as inevitably stifling a child’ emotional life. The film deconstructs the dichotomy between childhood and adulthood—unlike the anti-communist propaganda and the subsequent demonization of institutions in Goldberger's films, *Čekají každou neděli* takes a much subtler look at public care in terms of the child's experience. According to the film director, he wishes he had edited the film differently and downplayed the emotions in favour of a more thoughtful approach to the child's experience.^94^

**Figure 7.**
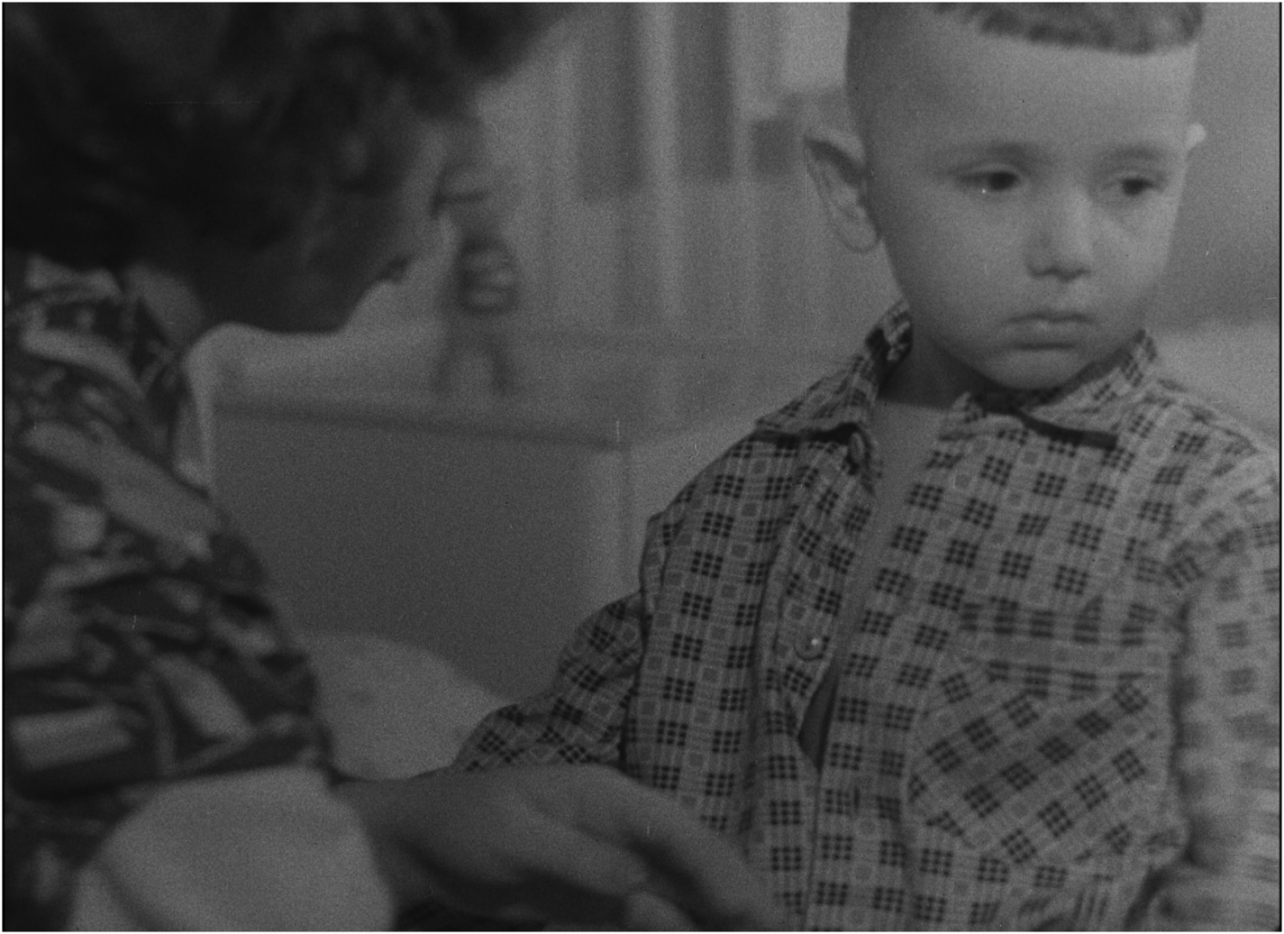
Children's subjectivity (*Čekají každou neděli*, 1962). *Source*: NFA.

**Figure 8.**
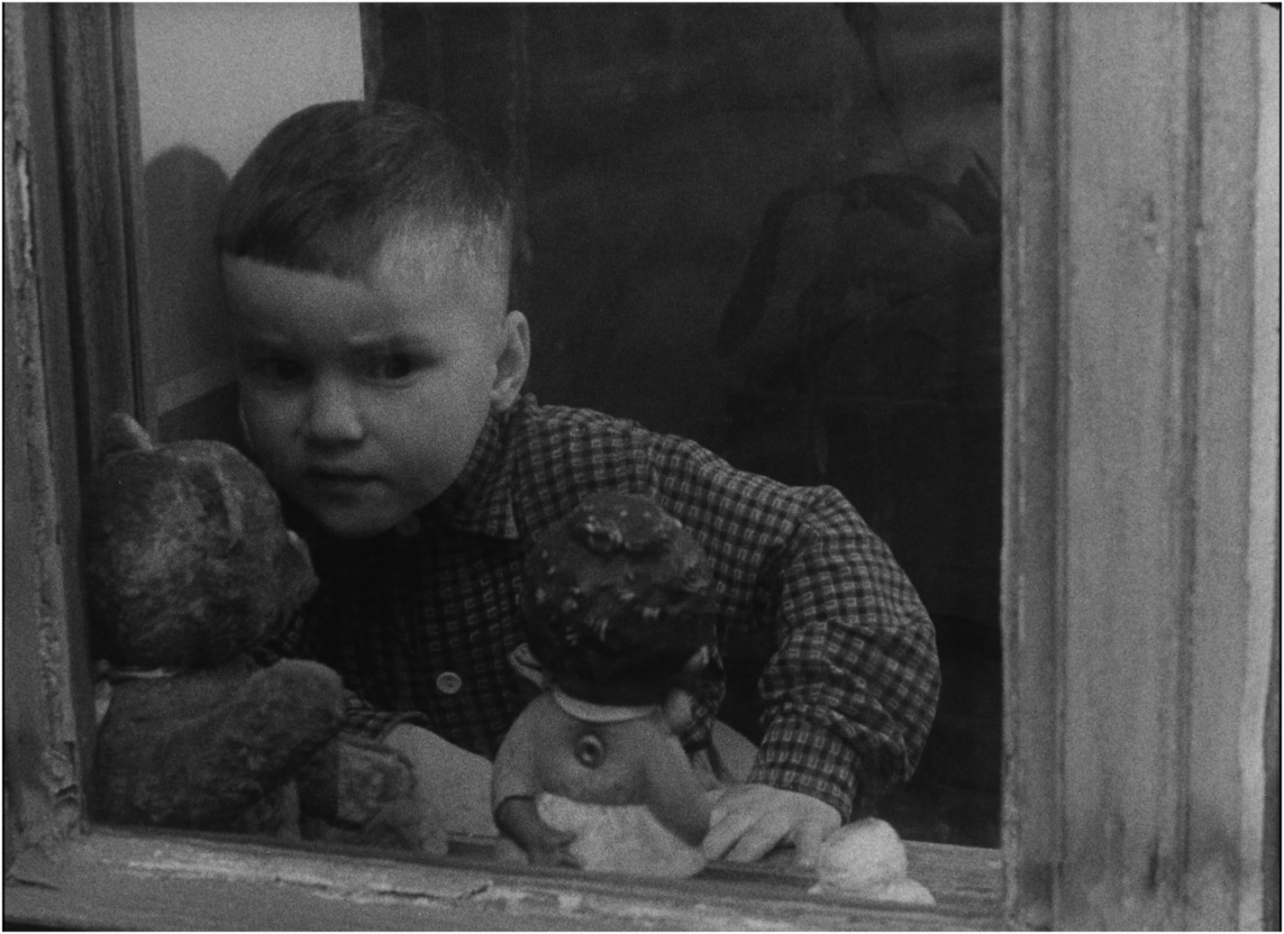
Children's subjectivity (*Čekají každou neděli*, 1962). *Source*: NFA.

**Figure 9.**
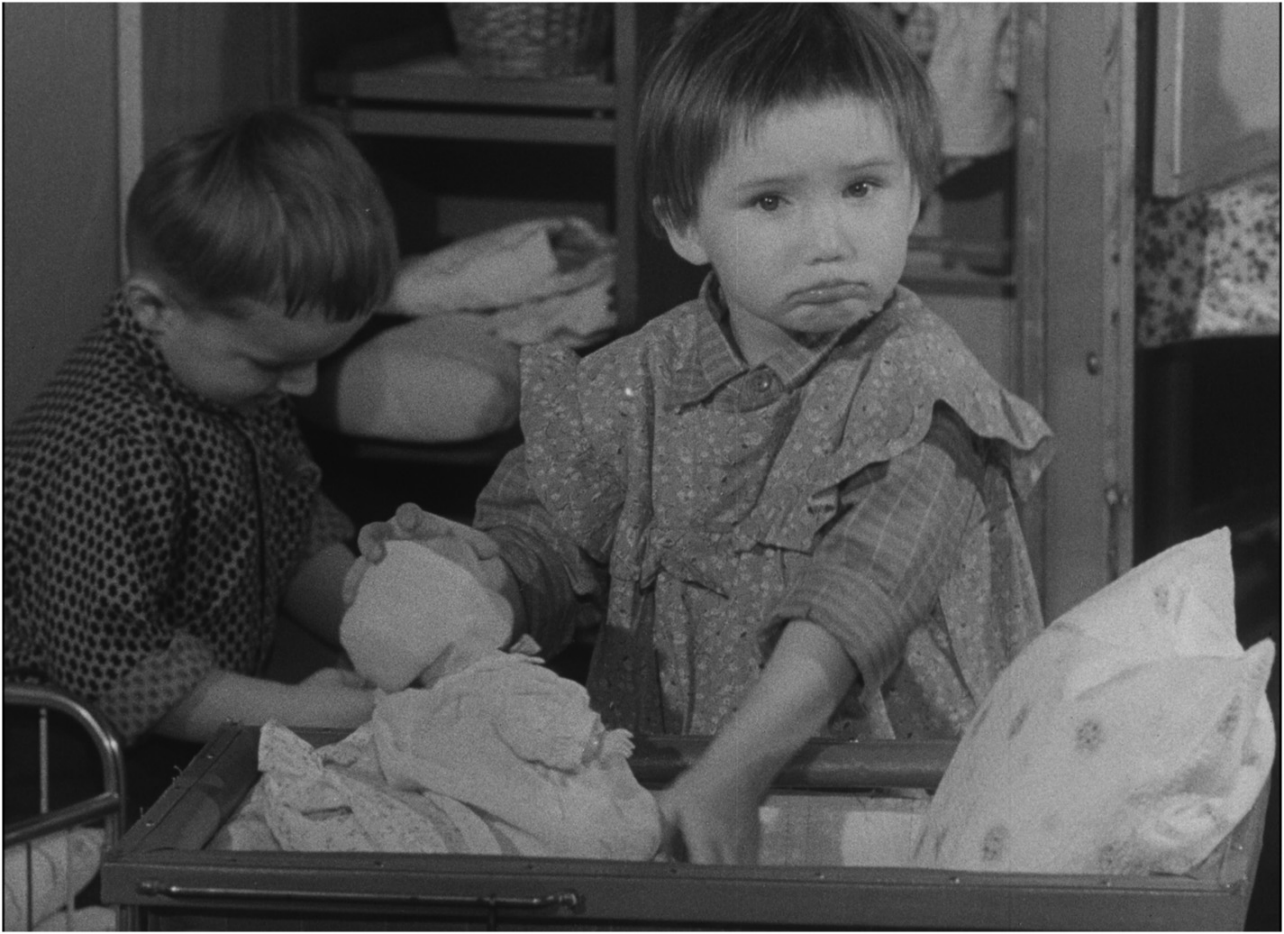
Children's subjectivity (*Čekají každou neděli*, 1962). *Source*: NFA.

**Figure 10.**
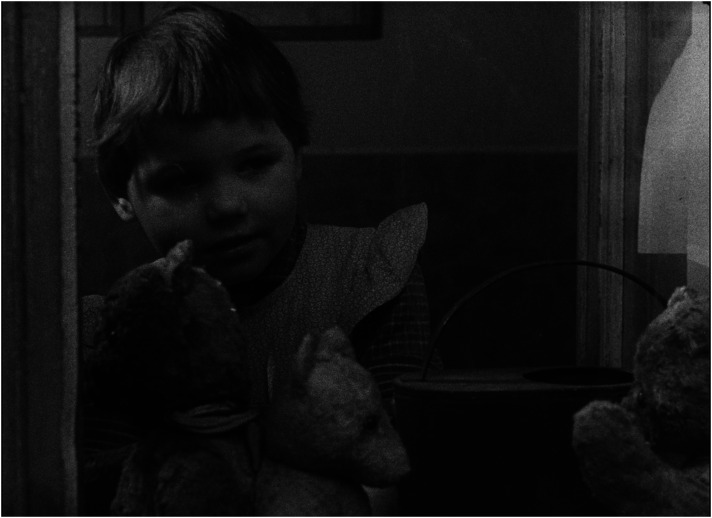
Children's subjectivity (*Čekají každou neděli*, 1962). *Source*: NFA.

The children in the film are not ‘examples’ aimed at illustrating the variety of typical reasons why children are placed, and remain, in institutions, rather they are children who do not like the space of the institution, who are envious of the other children lucky enough to be taken to a new home, and who experience mixed feelings when they are to be adopted (see Figures 7 to 10). The inevitable deprivation of children in institutions is conveyed through the consistent discrepancy between the rich inner life of the children and the limitations on their social space. For the most part, the children are shown in a static position: they eat, they play while sitting at the table, they look out the window. This forced deprivation becomes even more visible in the scene featuring a girl with limited ability to move, obviously because of disability, who shares her deepest desire to be with her parents, while caregivers help her dress. The film depicts active movement only in the two children who are taken to a family home.

**Figure 11.**
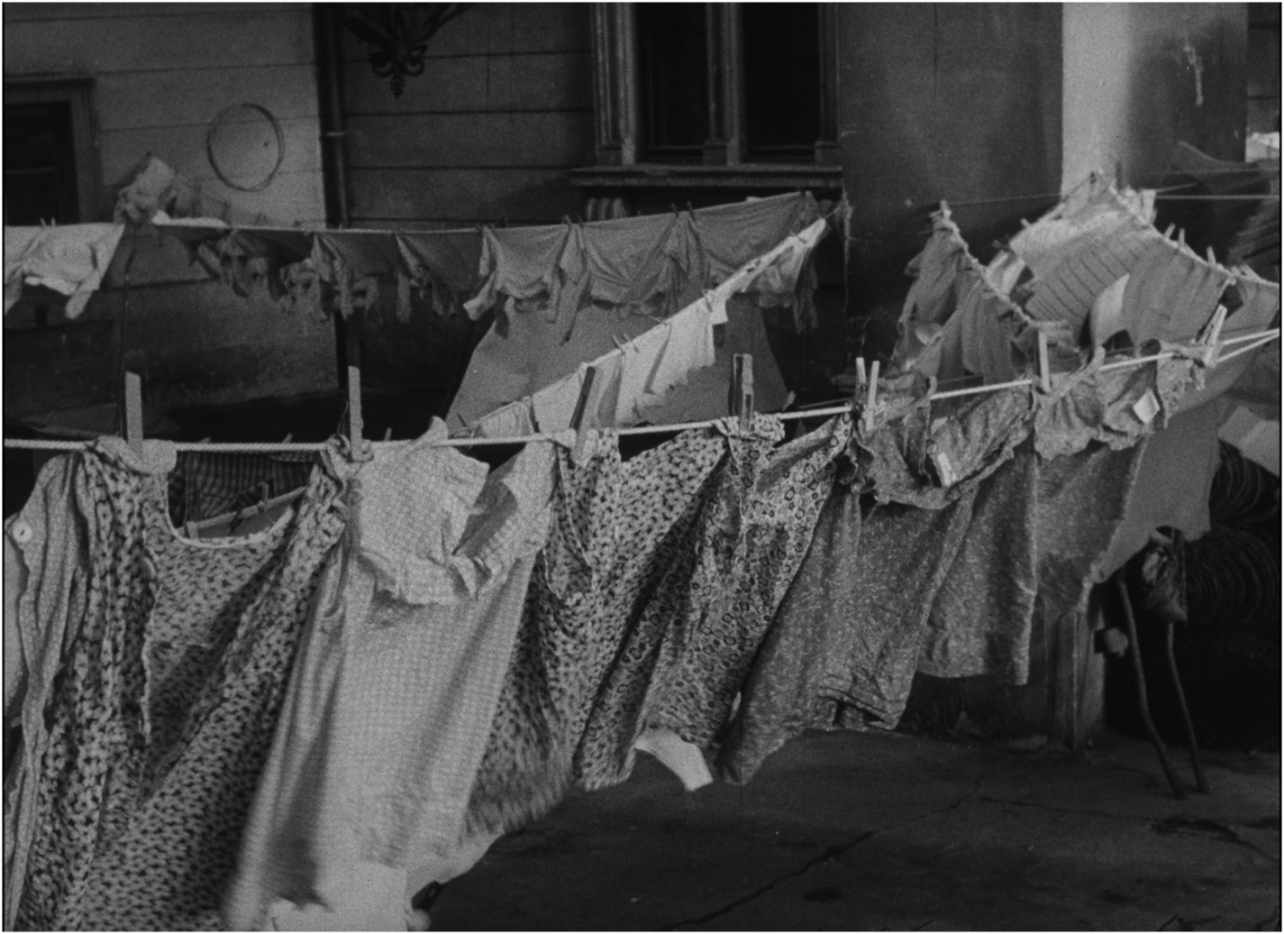
Emancipation of family and institution (*Čekají každou neděli*, 1962). *Source*: NFA.

**Figure 12.**
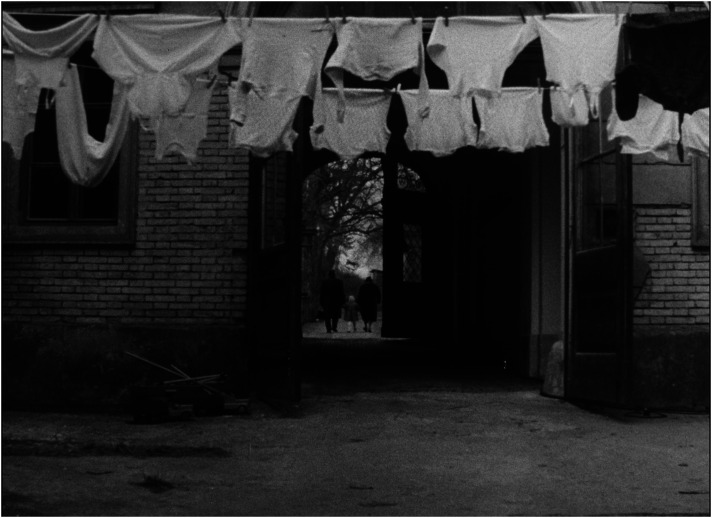
Emancipation of family and institution (*Čekají každou neděli*, 1962). *Source*: NFA.

The close-ups of the children's faces sharpen their individuality and contrast it with the univocal material world around them: at the beginning and at the end of the film there are images of underwear (which is the same for all the children) drying outside on a clothesline in a gusty wind—a primary visual trope for the feeling of impending deprivation for those who must remain in the institution (see Figures 11 and 12). As in *Dva stoly mezi námi*, in which the space of the courtroom produces a vacuum between former spouses, this metaphor of absence of any positive connection, opposing intentions of people and operation of institutions seems to be the main answer to the question of why only two of 56 children have been given the opportunity to be placed with a family. Each of the viewers has his or her own experience to fill this vacuum. This interactive effect of civic engagement was confirmed by feedback from viewers who reported: ‘In the film, we have the opportunity to hear directly from the children how they miss their parents, to make sure that although they have toys, clothes and a great bed in the orphanage, this is not enough for them’.^95^ However, the film did not reach a wide audience. It was mostly shown as an introduction to the Soviet films that were very unpopular among the Czechoslovak public in the 1960s.^96^

The call for civic emancipation became even more noticeable in *Dobrý člověk ještě žije*, the last documentary produced by Otta Bednářová^97^ before she was dismissed from Czechoslovak Television as ‘unreliable’ by the communist regime in 1970. In cooperation with Marie Šolleová—famous for her children's texts, including the plots for *Večerníček*, a show featuring popular TV bedtime stories—Bednářová updates the idea of collective solidarity around the mission to help Czechs find their way after political catastrophe and the beginning of ‘normalization’. Oppressive uncertainty and isolation, the pressure of which was unbearable, provide the emotional background to the narrative in the first few minutes of *Dobrý člověk.* Traditional preparations are underway in an apartment, decorated for the celebration of the main family holiday for most Czechs, Christmas Eve, one of the first after the Soviet invasion. But the woman in the apartment remains alone. Each preparation reminds the viewer of the choice Czechs faced after the invasion: leave or stay, submit or resist, and most importantly, hope or despair.

This deliberately controversial picture sets the tone for the entire film, which not only builds a strategy of resistance in an uncertain situation but operates as a counternarrative to pre-1968 attempts to disseminate family care for children, which had developed in the 1960s. Coping with invasion's experience is obviously a main motivating force for radicalizing the relation to ‘the past, which is transformed into that which lives and breathes in the present’.^98^

Even the title makes viewers think about the opposition between collective identity and individual responsibility or *Dobrý člověk* versus *Lidé*. The film title reproduces the Czech translation of the title of the novel *Colas Breugnon* by Romain Rolland, which became extremely popular after František Laurin made it into a film in 1967. In the finale of *Dobrý člověk*, the audience reads a quote by Rolland: ‘I recognize the only sign of nobility, and that is kindness’. Clearly, the coherent intertextuality of the documentary, the Rolland's novel and its filmed version works to promote the main idea of the need to live through difficult times, as did Colas Breugnon, an ordinary French carpenter, by practising solidarity.

The film brings into focus the agency of a younger generation who should take a leading role in the survival of the nation. Every story about a kind person in the film is a story about a kind woman, mostly young, or even a group of girls. High school girls help lonely elderly people; a young woman in her 30s helps an elderly neighbour with a visual impairment. The film pays special attention to the family placement of children with special needs—26 minutes of the 45-minute film are dedicated to three cases of helping children from institutions through family hosting during weekends and holidays.

This part of the film opens with the story of Zdeněk Makuňa, a Romani boy with a visual impairment, who is taken in for weekends and holidays by Mrs. Lašková, a kindergarten director and a mother of three children. The Laškov family is accustomed to taking in the children no one wants, to show them what it means to have family or ‘to show what irresponsible parents deprived them of’. In contrast to *Lidé*, *Dobrý člověk* depicts family proximity not through joy and play but by uniting different generations in difficult times. Lašková explains her intention to help children through her experience as an educator, or even a representative of biopower: ‘I worked in a residential weekly kindergarten and saw how sad it was for the children who stayed overnight, how they constantly called out for their mother’. The film opposes the official position of prioritizing public care for children with disabilities and civic initiatives, albeit not directly, but consistently. Not institutions or experts but families and a close community serve as the main resource for helping these children. This motif develops further in the next two cases.

Maria, a student at the pedagogical college, takes in a 3-year-old boy she has met during her field training. As in *Čekají každou neděli*, viewers follow the disappointment of the other children, who are not lucky enough to have somebody willing to take them home. But Maria is shown in her college, surrounded by peers, and there is no hint that her engagement is a kind of exception to the rule. Her family and neighbours totally support her intention to participate in the upbringing of little Ladik.

Similarly, Agnes, a 23-year-old nurse from an infant home, becomes attached to Jiří, a 3-month-old boy in her care, and continues to take him home to her parents even after he is transferred from the infant home to the orphanage—for more than 4 years. Like Maria, she receives an enormous amount of support from her parents, who have even started to take in Jiří's siblings. Neither Maria nor Agnes discusses their vocational training as a resource that helps them to care for the child—they are just young girls with an open heart, ready, despite their youth and attractiveness, to spend time at weekends with a baby left without a family. In this manner, the message of *Dobrý člověk* contests the message conveyed by the proponents of attachment theory, which stresses the role of experts and theoretical preparation in educating children.

In *Lidé*, the main part of the action takes place in family spaces. By contrast, the action in *Dobrý člověk* mainly takes place in public institutions—a pedagogical college, a boarding school, an orphanage and an infant home. Mrs. Lašková, Adela and Maria are constantly on the road, picking up the children from the institutions and bringing them home. This obvious difference reflects not only the filmmakers’ view that family stability was lost after the Warsaw Pact invasion but also the need to move from family care to community care based on informal networks around children. This is seen as necessary for the survival of the nation, and is embodied by three boys totally dependent on women. The film seems to move, throughout, towards a negation of patriarchal order without any prospect of achieving this.

## 3. Conclusion

At the heart of the Czechoslovak politics of family care for children with disabilities, early childhood was ‘emblematic of the politics of life because of the centrality of development discourse’.^99^ The central mechanism that triggered changes in child placement practices since the 1950s was the introduction of the concept of ‘deprivation’, which fundamentally reconfigured previous hierarchies of children, families and institutions themselves within practices such as residential care and substitute family care.

Having become central to the revision of the criteria for a ‘proper’ childhood, the concept of deprivation introduced such criteria as the duration of the stay at the institution, the experience of emotional neglect and the proximity between children and adults. Each of these criteria operated against family placement of children with disabilities, one of the target groups of long-term public care, whose emotional development was limited by the predominance of medical care, and who were often assumed to be limited in their ability to develop affection.

In public discourse, Deprivation Theory reinforced the dominant narratives about the interrelation between family care and child development. These had been translated into general cultural expectations and constituted a sense of normality rooted in patriarchal norms.^100^ Reproducing this narrative approach not only explains the long-term acceptance of Goldberger's films but calls for a consistent historicization of parenting children with disabilities in the form of counter-narratives that question ableism and patriarchy.^101^ The films by Táborský and Bednářová that connect family placement with civic emancipation and local solidarity—as in the recently published diary by Hana Kulasová, the mother of a child with an autistic disorder who resisted the pressure of medical experts and kept her son in family education^102^—enable a better understanding of how critical historicization can help to overcome the cruel optimism of the patriarchal view of family and family placement, something that public policy still needs to address.

## Notes

1. In Czech, *náhradní rodinná péče* (substitute family care) is an umbrella concept covering both adoption and foster care as alternatives to efforts aimed at reuniting the child with biological family.

2. There is a long tradition of invalidating Romani children as those who become disabled because of a vulnerable environment, which results in multiple practices of discriminating against Romani people.

3. Ochránce lidských práv, ‘Stanovisko k rovnému zacházení s dětmi při zařazování do evidence náhradní rodinné péče’, V Brně dne 28.4.2010 Sp. zn.: 1825/2010/VOP/AHŘ.

4. V. Kuchařová, *Zhodnocení a optimalizace řízení systému sociálně-právní ochrany (ohrožených) dětí a rodin ve vybraných regionech*, Praha 2010, 171.

5. Ochránce lidských práv ‘Stanovisko’.

6. V. Shmidt, ‘Czech Child Protection after 1989: Between Socialist Legacy and the European Call for Democratic Legitimacy’, in: J. Berrick / N. Gilbert / M. Skivenes (eds.), *International Handbook of Child Protection Systems,* Oxford 2023, 422–443.

7. J. Cousins, ‘Disability: Still Taboo in Family Placement?’, in: *Adoption and Fostering 33* (2009) 2, 54–65.

8. H. Keller, *The Myth of Attachment Theory: A Critical Understanding for Multicultural Societies*, London 2021.

9. B. Pierik, ‘Patriarchal Power as a Conceptual Tool for Gender History’, in: *Rethinking History* 26 (2022) 1, 71–92, 83.

10. E. White / R. Mistry, ‘Parent Civic Beliefs, Civic Participation, Socialization Practices, and Child Civic Engagement’, in: *Applied Developmental Science* 20 (2016) 1, 44–60.

11. B. Bradley, ‘From Biosociality to Biosolidarity: The Looping Effects of Finding and Forming Social Networks for Body-focused Repetitive Behaviours’, in: *Anthropology & Medicine* 28 (2021) 4, 543–557.

12. V. Welch et al., ‘Permanence for Disabled Children and Young People through Foster Care and Adoption: A selective Review of International Literature’, in: *Children and Youth Services Review* 53 (2015), 137–146.

13. G. Hunnicutt. ‘Varieties of Patriarchy and Violence against Women: Resurrecting ‘Patriarchy’ as a Theoretical Tool’, in: *Violence Against Women 15* (2009) 5, 553–573, 555.

14. S. H. Franzblau, ‘Historicizing Attachment Theory: Binding the Ties that Bind’, in: *Feminism and Psychology* 9 (1999) 1, 22–31.

15. H. Havelková, ‘‘Patriarchy’ in Czech Society’, in: *Hypatia* 8 (1993) 4, 89–96, 94.

16. K. Lišková, *Sexual Liberation, Socialist Style: Communist Czechoslovakia and the Science of Desire, 1945–1989*, Cambridge 2018, 164–171; H. Hašková / R. Dudová, ‘Selective Pronatalism in Childcare and Reproductive Health Policies in Czechoslovakia’, in: *The History of the Family* 25 (2020) 4, 627–648; S. Saxonberg / L. Formánková / H. Hašková, ‘The Role of Experts in Forming Family Policy under an Adversarial Subsystem in the Czech Republic’, in: *International Journal of Social Welfare* 32 (2023) 1, 101–112.

17. L. Chelcea / O. Druţǎ, ‘Zombie sScialism and the Rise of Neoliberalism in Post-socialist Central and Eastern Europe’, in: *Eurasian Geography and Economics* 57 (2016) 4–5, 521–544, 522.

18. R. Dinu, ‘Recent Historiographical Trends in Scholarship on Disability and Socialism in Eastern Europe’, in: *Scandinavian Journal of Disability Research* 24 (2022) 1, 42–53.

19. M. Savelli, ‘Beyond Ideological Platitudes: Socialism and Psychiatry in Eastern Europe’, in: *Palgrave Communications* 4 (2018) 45, https://doi.org/10.1057/s41599-018-0100-1; R. H. Dinu / S. Bengtsson (eds.), *Disability and Labour in the Twentieth Century: Historical and Comparative Perspectives*, London 2023.

20. Contesting views on such pathways can be found in J. Rákosník / R. Šustrová, ‘'State Socialist Biopolitics ‘Four Stages of Human Development in Post-War Czechoslovakia’’, in: B. Klich-Kluczewska / J. von Puttkamer / I. Rebitschek (eds.), *Biopolitics in Central and Eastern Europe in the 20th Century: Fearing for the Nations*, London 2022, and V. Shmidt, *Politics of Disability in Interwar and Socialist Czechoslovakia: Segregation in the Name of the Nation*, Amsterdam 2019.

21. D. Vargha, *Polio across the Iron Curtain: Hungary's Cold War with an Epidemic*, Cambridge 2018; A. Antić, ‘Decolonizing Madness? Transcultural Psychiatry, International Order and Birth of a ‘Global Psyche’ in the Aftermath of the Second World War’, in: *Journal of Global History* 17 (2022) 1, 20–41.

22. One of the rare examples of applying such an approach is a contribution by P. Schmüser, ‘‘We as parents must be helped.’ State–Parent Interactions on Care Facilities for Children with ‘Mental Disabilities’ in the GDR’’, in: K. Kolářová / M. Winkler (eds.), *Re/imaginations of Disability in State Socialism: Visions, Promises, Frustration*, Frankfurt, New York 2021, 215–258.

23. Národní Archiv (NA), The Fond of the Ministry of Health, Care for children (MZD, LP).

24. Z. Kozáková, ‘Proměny v časopise Vlasta v letech 1988-1991’, Master Thesis Charles University Prague 2021; M. Kolková, ‘Proměna zobrazování žen v časopisu Vlasta, Master Thesis. MUNI Department of Media Studies and Journalism, Brno 2021.

25. M. Faßig, ‘Discourses of Prevention, Risk and Responsibility in the Women's Magazine *Vlasta* (1950s–1980s)’, in: Kolářová / Winkler (eds.), *Re/imaginations of Disability*, 149–164.

26. B. Baird, ‘Child Politics, Feminist Analysis’, in: *Australian Feminist Studies* 23 (2008) 57, 291–305, 291.

27. Ibid., 296.

28. L. Česálková, *Atomy věčnosti. Český krátký film 30. až 50. Let*, Praha 2014.

29. F. Jameson, *The Political Unconscious,* London 1981.

30. S. Ahmed, *The Cultural Politics of Emotion*, Edinburgh 2014.

31. L. Berlant, *Cruel Optimism*, Durham 2011, 52.

32. Ibid., 1.

33. Ibid., 2

34. Vyhlaška ministra ochrany práce i pracovních sil 316 1951 Uř. 1 Převod domovů pro děti do oboru působnosti Ministerstva zdravotnictvi NA, MZD, LP.

35. ‘Nařízení ministra zdravotnictví čis. 94: Dětské domovy pro dětí od 1 roku do 3 let’, 1952 NA, MZD, LP

36. After 1945, *pěstounky* (caregivers) and *pomocné pěstounky* (assistant caregivers) were mostly recruited among young women from the most vulnerable social groups. Along with training, these women received social support, including accommodation and other benefits, while nurses occupied a much more privileged position.

37. Ministerstvo zdravotnictví ‘Organizační a provozní řad dětských domovů 31-220 9011: Příloha 2 k oběžníku’, 1952 NA, MZD, LP.

38. V. Shmidt, *Politics of Disability in Interwar and Socialist Czechoslovakia: Segregation in the Name of the Nation*, Amsterdam 2019, 119.

39. ‘Usnesení příjaté na instruktáži krajských pediatrů, krajských dětských a jeslových sester dne 26. listopadu 1954 v Praze’ NA, MZD, LP

40. A. Heinrich, ‘The Emergence of the Socialist Healthcare Model After the First World War’, in: F. Nullmeier / D. González de Reufels / H. Obinger (eds.), *International Impacts on Social Policy. Global Dynamics of Social Policy*. Bern 2022.

41. Radio Free Europe (RFE) ‘Kindergarten Attendance Obligatory 12 December 1951 IX – 2235’ OSA 300-30-3 box 69.

42. RFE, ‘Neglected children in čSR: Children's homes Item No 686/54 K/D 27 Jan VIII/W/7345’, Open Society Archive (OSA) 300-30-3 box 69.

43. RFE, ‘Conditions in Kindergartens at Aš 27 September 1951 I/420 the source’, OSA 300-30-3 box 69, p.2.

44. RFE was established in 1949 by the Central Intelligence Agency as a vehicle for anti-communist propaganda. From the very beginnings of its operation, Czech journalists played an exceptional role in the development of RFE's broadcast content; more in A. Grutza, ‘Radio Free Europe and Cold War *Truth Games*: Trustworthy Messages Beyond the Great Divide’, in: *Historical Journal of Film, Radio and Television*, 39 (2019) 3, 479–498.

45. RFE, ‘Conditions in Kindergartens’, 2.

46. RFE, ‘Conditions in Kindergartens’, 4.

47. RFE ‘Evaluation and Analysis Department: Czechoslovak Unit News Background: The Future of the Czechoslovak family’, 1960 OSA 300-30-3 box 75.

48. J. M. H. Lindbeck, ‘Communist Policy and the Chinese Family’, in: *Far Eastern Survey*, 20 (1951) 14, 137–141

49. M. Blaive, ‘The Cold war? I Have It at Home with My Family’: Memories of the 1948–1989 Period beyond the Iron Curtain,’ in: K. H. Jarausch / Ch. Ostermann / A. Etges (eds.), *The Cold War: Historiography, Memory, Representation*, Berlin 2017, 203–223.

50. ‘Usnesení příjaté na instruktáži krajských pediatrů’.

51. ‘Směrnice X..sjezdu KSČ pro rok 1955: O zlepšení péči o dítě v jeslích, dětských domovech a zemědělských útulcích a prohloubení zdravotní služby’, NA, MZD, LP.

52. Státní úřad stistický republiky československé, *Statistická ročenka republiky československé* 1957 Praha 1957, 221; Státní úřad stistický republiky československé, *Statistická ročenka československé socialistické republiky 1964,* Praha 1964, 411.

53. Marie Damborská (1913–1991) was chief paediatrician in the infant home in Luhačovice and one of the founders of early intervention in Czechoslovakia.

54. Antonín Mores (1908–1997) was a famous paediatrician who led the infant home in Olomouc.

55. Ladislav Zeman (1917–2016) started his career in Svitavy as the chief paediatrician in the infant home and continued his career in Prague.

56. These efforts started to be promoted in the socialist media at the end of the 1950s; see Zuzana Kavanová, in her short review, ‘Z lásky k životu’ (*Rovnost*, 21. December 1957), described the importance of the reproduction of family space for further family placement of those children placed into infant homes.

57. A. Prokešová, ‘Z dětských domovů’, in: *Vlasta* 18, 15. January 1964, 3.

58. M. Trubač, ‘Kde mají domov?’, in: *Vlasta* 18, 1. January 1964.

59. F. Křivanek, ‘Význam vytváření rodinného prostředí a systém rodinných buněk v úSP’, in: Ministerstvo práce a sociálních věci ČSR, *Sborník přednášek z celostátního školení vychovatelů v ústavech sociální péče pro duševní postiženou mládež,* Praha 1972, 1–13, 4.

60. Jíři Dunovský (1930–2015) was a founder of the Social Paediatrics Research Laboratory, Children's Crisis Center, Society of Social Paediatrics in the Czech Republic and other facilities and institutions for helping vulnerable children. Since the 1970s, he was one of the UNESCO experts responsible for developing strategies for children without sustainable parental care.

61. Ahmed, *The Cultural Politics*, 127.

62. J. Langmeier, ‘Rodinné vztahy a socializace dítěte’ [Family relationship and child's socialization], in: Ministerstvo práce a sociálních věci ČSR, *Sborník přednášek*, 100–134, 110.

63. Z. Matějček (1922–2004) was a child psychologist, one of the emblematic socialist experts in the field of family care for children.

64. J. Langmeier (1921–2007) was a clinical psychologist who gained a reputation within the field of psychometry.

65. J. Langmeir, Z. Matějček, *Psychická deprivace v dětství,* Praha 1963, 219–222.

66. Z. Matějček, ‘Indikace jednotlivých form náchradní rodinné péče’, in: Ministerstvo práce a sociálních věci ČSR, *Metodika odborných služeb v náhradní rodinné péčí*. Praha 1979, 60–86, 66–67.

67. Z. Matějček, ‘K otázce adopcí’, in: *Československá pediatrie*, 18 (1963) 1, 842–846.

68. Prokešová, ‘Z dětských domovů’.

69. Matějček, ‘Indikace jednotlivých form’, 62.

70. Ministerstvo práce a sociálních věci ČSR, *Metodika odborných služeb*, 11.

71. M.Sedloň, ‘Mateřské havárie’, in: *Vlasta* 15, 13 April, 1968, 4–9.

72. Even publications aimed at presenting initiatives for helping biological parents were dramatically titled: ‘Victims of their own parents?’: Obete vlastných rodičov?, *Nové slovo* 12. March 1970, 2.

73. J. Paleček, *Osvojování dětí v České republice: Zpráva z výzkumu v České republice na téma osvojení*, Praha 2017.

74. ‘Paragrafy mezi rodinou a dětmi’, in: *Vlasta* 17, 20 March 1963.

75. J. Bennett, *History Matters: Patriarchy and the Challenge of Feminism*, Pennsylvania 2007, 77.

76. Pierik ‘Patriarchal Power’, 73–74.

77. F. Jameson, *The Political Unconscious: Narrative as a Socially Symbolic Act*, Cornell 1982, 108.

78. ‘Kratká bilance krátkého filmu’, in: *Květy,* 25 April 1964, 34.

79. V. Bystrov, ‘Film má vždy přijít včas’: rozhovor s Kurtem Goldbergerom’, in: *Kino* 19, 4, 27 February 1964.

80. P. Holland, *Picturing Childhood: The Myth of the Child in Popular Imagery*, London 2004, 15.

81. Bystrov, ‘Film’.

82. Peter John Frank (1934–2013) was a lecturer in Soviet history at the University of Leeds, later professor of Russian politics at the University of Exeter and popular media commentator on Russian affairs, who contributed to several BBC broadcasts between the 1970s and 1990s.

83. P. J. Frank, ‘Social Policy’, in: *Listener,* 25 May 1967, 4.

84. ‘Tři děti hledají rodiče’, in: *Vlasta* 17, 20 March 1963, 2.

85. V. Hajková. ‘Tři děti našly rodiče’, in: *Vlasta* 41, 9 October 1963, 41.

86. Ahmed, *The Cultural Politics.*

87. Berlant, *Cruel Optimism*, 441

88. J. C. Garlen / J. A.Sandlin, ‘Happily (n)ever after: The Cruel Optimism of Disney's Romantic Ideal’, in: *Feminist Media Studies* 17 (2017) 6, 957–971, 963.

89. J. Dunovský a L. Zeman, ‘Sociální situace dětí propuštěných z Kojeneckého ústavu v Praze (výzkum po 14 – 19 letech)’, in: *Sociologický Časopis* 12 (1976) 6, 665–671.

90. S. Vertoont et al, ‘Un/recognisable and Dis/empowering Images of Disability: A Collective Textual Analysis of Media Representations of Intellectual Disabilities’, in: *Critical Studies in Media Communication* 39, 2022 1, 1–14.

91. Berlant, *Cruel Optimism*, 51.

92. Personal communication with V. Táborský, 23 October 2022.

93. M. Šrajer, ‘Václav Táborský’ available online https://www.filmovyprehled.cz/cs/revue/detail/vaclav-taborsky.

94. Personal communication with V. Táborský, 23 October 2022.

95. V. Zezuláková, ‘Dětí touži po domove’, in: *Vlasta* 17, 16 January 1963.

96. Personal communication with V. Táborský, 12 November 2022.

97. Otta Bednářová (1927) is a journalist with a reputation for being a die-hard fighter against communist authorities. During Normalization, Bednářová took such an active position in the dissident movement that she was sentenced to 3 years without parole: J. Cysařová, *Já prostě nemohu žít jinak: česká publicistka Otka Bednářová*, Praha 2010.

98. Ahmed, *The Cultural Politics*, 180.

99. K. Wells, ‘The Politics of Life: Governing Childhood’, in: *Global Studies of Childhood* 1 (2011) 1, 15–25, 18.

100. M. Bamberg, ‘Considering Counter Narratives’, in: M. Bamberg / M. Andrews (eds.), *Considering Counter Narratives: Narrating, Resisting, Making Sense,* Amsterdam 2004, 351–371.

101. P. Lalvani / L. Polvere, ‘Historical Perspectives on Studying Families of Children with Disabilities: A Case for Critical Research’, in: *Disability Studies Quarterly* 36 (2016) 4, available online: https://dsq-sds.org/article/view/3209/3291.

102. H. Kulasová, *Kapka kámen vyhloubí: Deník odvážné mamy o autismu a socialismu,* Praha 2021.

